# *Arabidopsis thaliana* Roots Exposed to Extracellular Self-DNA: Evidence of Epigenetic Effects

**DOI:** 10.3390/epigenomes9020013

**Published:** 2025-04-30

**Authors:** Alessia Ronchi, Guido Incerti, Emanuele De Paoli, Speranza Claudia Panico, Giovanni Luca Sciabbarrasi, Pasquale Termolino, Fabrizio Cartenì, Mariachiara Langella, Maria Luisa Chiusano, Stefano Mazzoleni

**Affiliations:** 1 Department of Agricultural, Food, Environmental and Animal Sciences, University of Udine, Via delle Scienze 206, 33100 Udine, Italy; alessia.ronchi@unipd.it (A.R.); emanuele.depaoli@uniud.it (E.D.P.); speranza.panico@uniud.it (S.C.P.); giovanni.sciabbarrasi@uniud.it (G.L.S.); 2 Department of Agronomy, Food, Natural Resources, Animals and Environment, University of Padova, Via dell’Università 16, 35020 Legnaro, Italy; 3 National Research Council, Institute of Biosciences and Bioresources, 80055 Portici, Italy; pasquale.termolino@ibbr.cnr.it; 4 Department of Agricultural Sciences, University of Napoli Federico II, Via dell’Università 100, 80055 Portici, Italy; fabrizio.carteni@unina.it (F.C.); mariachiara.langella@unina.it (M.L.); chiusano@unina.it (M.L.C.)

**Keywords:** self-DNA inhibition, whole genome bisulfite sequencing (WGBS), RNA-seq, plant early stress response, differentially methylated regions (DMRs) analysis

## Abstract

Background: Previous evidence demonstrated DNA methylation changes in response to stress in plants, showing rapid changes within a limited time frame. Exposure to self-DNA inhibits seedling root elongation, and it was shown that it causes changes in CG DNA methylation in *Lactuca sativa*. We assessed cytosine methylation changes and associated gene expression patterns in roots of *Arabidopsis thaliana* Col-0 seedlings exposed to self-DNA for 6 and 24 h. Methods: We used whole genome bisulfite sequencing (WGBS) and RNA-seq analyses to assess genomic cytosine methylation and corresponding gene expression, respectively, on DNA and RNA extracted with commercial kits from roots exposed to self-DNA by an original setup. Fifteen hundred roots replicates, including the control in distilled water, were collected after exposure. Sequencing was performed on a NovaSeq 6000 platform and Ultralow Methyl-Seq System for RNA and DNA WGBS, respectively. Results: Gene expression in roots exposed to self-DNA differed from that of untreated controls, with a total of 305 genes differentially expressed and 87 ontologies enriched in at least one treatment vs. control comparison, and particularly after 24 h of exposure. DNA methylation, particularly in CHG and CHH contexts, was also different, with hyper- and hypomethylation prevailing in treatments vs. controls at 6 h and 24 h, respectively. Differentially expressed genes (DEGs) analysis, Gene Ontology (GO) enrichment analysis, and differentially methylated regions (DMRs) analysis, provided an integrated understanding of the changes associated with self-DNA exposure. Our results suggest differential gene expression associated with DNA methylation in response to self-DNA exposure in *A. thaliana* roots, enhanced after prolonged exposure. Conclusions: Main functional indications of association between DNA methylation and gene expression involved hypomethylation and downregulation of genes related to nucleotide/nucleoside metabolism (ATP synthase subunit) and cell wall structure (XyG synthase), consistent with previous observations from metabolomics and physiological studies. Further confirmation of these findings will contribute to improving our understanding of the plant molecular response to self-DNA and its implications in stress responses.

## 1. Introduction

Epigenetic research in plants has uncovered crucial mechanisms that influence various biological processes, such as the silencing of repetitive DNA sequences and the regulation of transposable elements (TEs) [1]. TEs are parasitic DNA elements capable of copying themselves and inserting into new genomic locations, hence disrupting gene function [2]. In fact, much of the epigenetic regulation of gene expression arises from managing disruptive TEs that are inserted near genes [3]. In the model plant *Arabidopsis thaliana*, DNA methylation of TEs primarily functions to suppress their movement within the genome [4]. However, these methylated sequences can also influence the expression of neighboring genes, often leading to reduced expression, suggesting a negative relationship between gene activity and the presence of silenced TEs [4,5,6].

One important function of DNA demethylation in plants is the activation of genes in response to biotic or abiotic stress. This frequently occurs through demethylation of TEs located near the 5′ regions of genes [7]. Temporary loss of DNA methylation, particularly during stress-induced TE activation, can contribute to genetic variation and thus play a role in plant evolution [8,9]. Modifying specific methylation contexts may serve as an adaptive mechanism for regulating gene expression. Notably, non-CG methylation helps shield genes from the negative effects of nearby TEs [10]. For instance, “CHH islands”—regions of high CHH methylation found at euchromatin/heterochromatin boundaries, first identified in Zea mays—are thought to strengthen TE silencing by maintaining a boundary between the silenced chromatin of the TE and the active chromatin of adjacent genes [11,12].

Cytosine methylation can also occur in promoter regions and within the coding sequences of actively transcribed genes [13]. High levels of 5-methylcytosine (5mC) in CpG-rich promoter regions are typically linked to transcriptional repression. Conversely, CpG-poor regions show a more complex, context-dependent relationship between DNA methylation and gene expression [14]. Another form of methylation, known as gene body methylation (gbM), involves CG methylation within gene exons. This mark is heritable across generations in plants and may be shaped by natural selection [15]. Differently, CHH methylation is largely removed in the male germline of *A. thaliana* and re-established during embryogenesis [16], meaning its inheritance is limited to one or a few generations. The inheritance of CHG methylation is less understood; although it persists through gametogenesis [16], studies on epimutation lines in *A. thaliana* suggest that CHG methylation is not consistently passed on across generations [17]. Of the three methylation types in plants, CG methylation appears most reliably inherited and thus the most likely candidate for long-term epigenetic adaptation. Emerging research also shows that gbM correlates with gene expression patterns, though whether this correlation is causal remains uncertain.

DNA methylation holds particular relevance for evolution due to its direct effects on gene regulation and its indirect role in TE suppression and reactivation [18,19]. As the most extensively studied epigenetic modification in plants, DNA methylation can be stably passed down through both mitosis and meiosis [20,21,22,23]. Both genetic and epigenetic changes can drive heritable phenotypic variations in plants, which modulate various developmental processes—such as flowering time, senescence, and gametogenesis—through DNA methylation [2]. Although the exact function of environmentally induced methylation changes is not always clear [24,25], it is widely believed that DNA methylation can serve as a medium through which environmental signals influence gene expression, thereby contributing to phenotypic plasticity [26,27]. Environmental stress is one of the key factors affecting cytosine methylation, potentially influencing how plants regulate gene activity in response [28,29]. Often, these changes are temporary, and the methylation pattern reverts to its original state [30,31,32]. However, in some cases, stress-induced epigenetic modifications may be inherited. If such modifications persist and prove advantageous when offspring face similar environmental conditions, they may become adaptive. When epigenetic changes are accompanied by genetic alterations—such as TE insertions—they can give rise to stable or variable epialleles. Otherwise, methylation changes may naturally revert or lead to mutations through deamination of methylated cytosines.

There is wide evidence that DNA methylation changes in response to abiotic and biotic stresses in plants [33]. Vega-Muñoz et al. [34] suggested that self-DNA acts as a DAMP in plants and observed changes in CG DNA methylation levels as well as increasing production of secondary metabolites associated with defense responses to stress. Experimental evidence supporting self-DNA as a DAMP in plants involved in species-specific damage recognition has been recently reviewed [35]. Self-DNA can trigger ROS production, enhance cytoplasmic calcium and membrane depolarization, and activate MAPK cascades [36,37]. Moreover, exposure to self-DNA can elicit differential expression of defense genes, such as those related to the jasmonic acid signaling pathway and genes responsive to abiotic stress [35,38,39,40]. The mechanisms by which exogenous self-DNA can influence gene expression, and the involvement of epigenetic machinery are, however, still unclear.

The aim of this study was to extend the investigation of cytosine methylation changes across the whole genome in association with gene expression levels. In particular, we present a whole genome bisulfite sequencing (WGBS) and RNA sequencing (RNA-seq) analysis of nucleic acids extracted from sample roots of *Arabidopsis thaliana* seedlings after short-term exposure to extracellular self-DNA.

## 2. Results

### 2.1. RNA-Seq and Gene Expression in Treated vs. Control Samples

**Figure 1:**
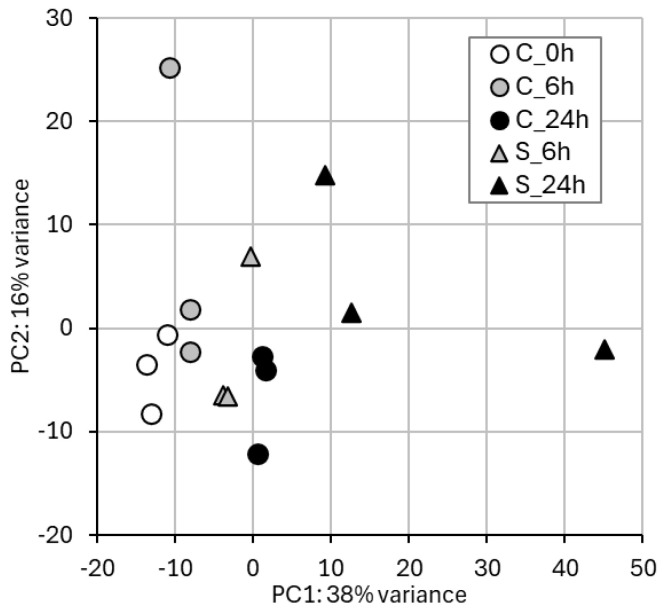
Results of PCA of gene expression data. Samples are generally clustered based on treatment type (C, control vs. S, self−DNA) and timing (0 h, 6 h, and 24 h) according to the variance explained by PC1 (38%) and PC2 (16%).

Mean RNA extraction yield over the 15 samples was 400 ng/mg of roots (fresh weight); Nanodrop ratios 260/280 and 260/230 were always >2, and average RIN (RNA integrity number) was 6.40, spanning between 5 and 8.50. At least 500 ng of RNA for each sample underwent RNA-seq analysis. PCA (Figure 1) highlighted that the groups of samples with similar gene expression levels corresponded to the treatment groups. In the bi-dimensional space defined by PC1 and PC2. In Supplementary Table S1, RNA-seq sequencing statistics for each sample are listed.

A clear, qualitative difference in gene expression between samples treated with self-DNA and control samples can be inferred by the PCA results. In particular, along PC1, a clear spreading of the samples is evident according to the interaction of two main conditions: (i) harvest timing (i.e., 0, 6, and 24 h), with early-harvested samples showing lower factorial scores compared to late-harvested samples within either treatment group (i.e., self-DNA exposed and controls); (ii) treatment group, with control samples consistently showing lower factorial scores on the PC1 as compared to the corresponding treated samples. In detail, samples treated with self-DNA for 6 h and relative controls were clearly separated. A more relevant difference in gene expression levels (Figure 1) was found between samples treated with self-DNA and the controls at 24 h, evident on both principal components.

**Figure 2:**
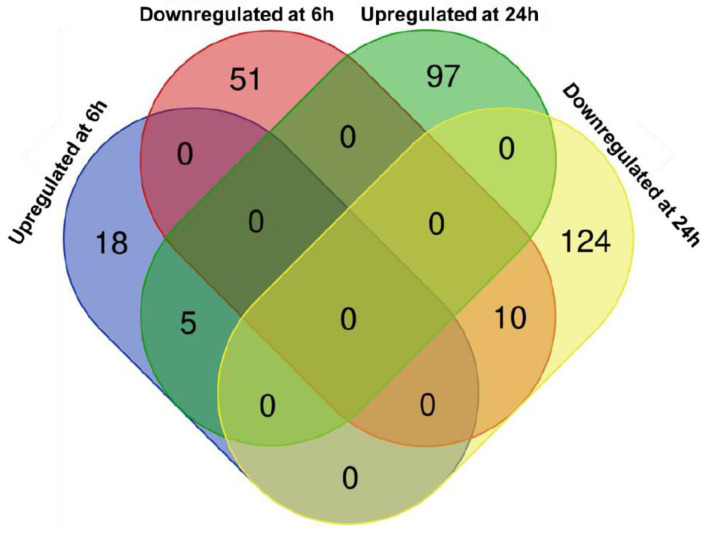
Venn diagram showing numbers of differentially expressed genes (DEGs) in each of the four groups (upregulated at 6 h, downregulated at 6 h, upregulated at 24 h, and downregulated at 24 h).

The Venn diagram in Figure 2 represents the 305 differentially expressed genes (DEGs) distinguishing between the upregulated (120 in total) and downregulated ones (185 in total), compared to the control at 6 (23 up and 61 down) and 24 h (102 up and 134 down) (Supplementary Datasets S1 and S2). It is evident that the number of DEGs is higher at 24 h compared to 6 h. At both time points, the downregulated genes are more abundant than the upregulated ones (~73% at 6 h and ~57% at 24 h). A small portion of the DEGs (15 genes) is shared between 6 and 24 h, and it reflects the proportion in favor of the downregulated genes.

### 2.2. Gene Ontology Enrichment

**Figure 3:**
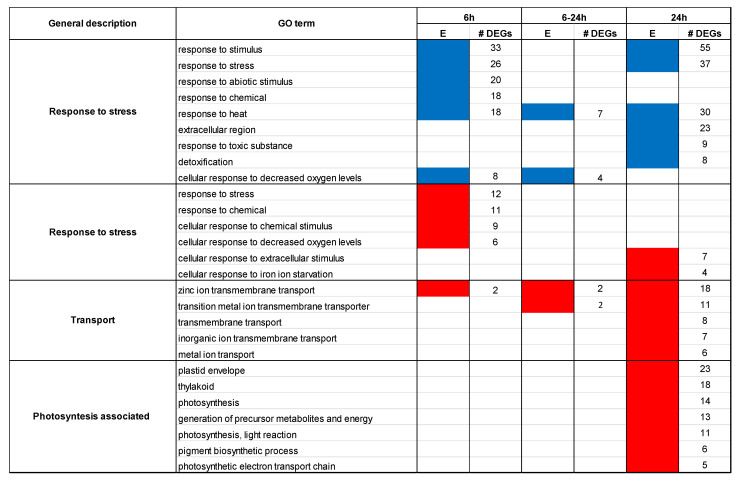
GO trends and general description. For each enriched GO term, enrichment by upregulated and downregulated genes (column E) is indicated in red and blue, respectively, for the comparisons control vs. exposed to self-DNA at 6 h. and 24 h. Counts of DEGs consistently downregulated or upregulated in both comparisons are also reported in the (6–24 h) column.

Figure 3 reports a summary of the Gene Ontology (GO) enriched in upregulated and downregulated genes in treatments vs. controls comparisons at either 6 h or 24 h. The number of DEGs in common in the two stages (“6 h–24 h”) is also considered. Different GO terms are grouped into one general category (e.g., “Response to stress”, “Transport”, etc.; Figure 3) when sharing the same set of enriching genes, while a complete list of all GO terms with detailed information is provided in Supplementary Dataset S3, as well as the detailed list of all DEGs in all comparisons in Supplementary Dataset S4. The analysis of downregulated genes after 6 h of exposure to self-DNA revealed enrichment of GO terms related to response to stimuli and stress, including abiotic stimulus, chemical, heat, and decreased oxygen levels (Figure 3). The specific GO “response to stimulus” (GO:0050896) includes 33 downregulated genes, which are also distributed among a further 22 associated GOs (from #1 to #22 in Supplementary Dataset S3). This means that the genes enriching the GO:0050896, being common to different GOs, can play roles corresponding to other different GO classes. As an example, 8 of those 33 genes (AT4G19520, AT5G41080, AT3G46230, AT4G10250, AT1G07400, AT1G59860, AT5G12020, and AT1G54050) are associated with GOs related to “response to hypoxia”, and 3 (AT5G45070, AT5G46470, and AT4G19520, of which AT4G19520 is in common with the previous group) are associated with “NAD+ nucleotidase” (Supplementary Datasets S3 and S4). Interestingly, out of the 33 downregulated genes associated with “response to stress” at 6 h some (AT1G07400, AT5G12020, AT1G54050, AT1G59860, AT5G59720, AT1G14780, AT4G37530, and AT4G35380) were still downregulated at 24 h (“6–24 h”, Figure 3, Supplementary Dataset S4), indicating a certain similarity in the response at the two time points. Out of these genes, 5 are HSP-like chaperone-encoding genes, while AT1G14780 is a MAC/perforin domain-containing protein, i.e., a component of the membrane attack complex. AT4G37530 belongs to the peroxidase protein superfamily, and AT4G35380 is a SEC7-like guanine nucleotide exchange family protein that encodes one of the functionally redundant ARF guanine nucleotide exchange factors involved in the endomembrane trafficking (Supplementary Dataset S4). Downregulation of “stress response to stimuli” at 24 h involved a much larger number of genes (55, Figure 3). In addition, downregulation of response to abiotic stimulus and chemicals was released, while downregulation of extracellular region, response to toxic substances, and detoxification appeared (Figure 3).

Considering the GO terms enriched by upregulated genes at 6 h, several GO terms were the same as those associated with downregulated genes at the same time point. This highlights the complex interplay between downregulated and upregulated genes associated with stress response and response to chemical and hypoxia gene ontologies (Figure 3), as a given functional response pattern can arise from both upregulated and downregulated genes annotated with the same set of GOs, depending on the stimulatory/inhibitory effect of each single gene product. In addition, the enrichment of GO terms associated with zinc ion transmembrane transport at 6 h in the biological process category (GO:0071577) and in the molecular function one (GO:0005385) was observed due to the upregulation of two genes, AT3G12750 and AT1G10970 (Supplementary Datasets S3 and S4), that are both zinc transporters playing important roles in zinc homeostasis and transport. Considering the GO terms enriched at 24 h by upregulated genes, those associated with zinc transmembrane transporters were still present and involved a larger number of genes and additional GO terms related more in general to metal and ion transport (Figure 3). Among the upregulated genes, five are proteins associated with zinc transport and uptake (AT3G12750, AT1G10970, AT1G05300, AT2G32270, and AT4G33020) (Supplementary Dataset S4). Finally, the photosynthesis light reaction (GO:0019684) as categories from Biological Processes (BP) were enriched in upregulated genes with “chloroplast” (GO:0009507) and “plastid” (GO:0009536) as main GOs from cellular component (CC).

### 2.3. Differentially Expressed Genes (DEGs)

To better address the DEGs functional analysis, we grouped the genes according to their functions in 7 classes (Table 1 and Supplementary Dataset S4): 1. Stress Response and Defense; 2. DNA/Protein Processing and Folding; 3. Growth and Development; 4. Transport, Signaling, and Homeostasis; 5. Energy, Metabolism, and Biosynthesis; 6. Gene Expression and Transcription Regulation; and 7. Uncharacterized and Miscellaneous Roles. This permits organizing upregulated and downregulated genes in the two stages for a more appropriate interpretation of the gene expression variability resulting from the analysis. The functional overlap among different classes forced the presence of few genes in more classes. We observed a downregulation of stress response genes, including 5 heat shock proteins (HSPs) (5 specific to the 6 h treatment and 5 in common at 24 h, plus 7 specific at 24 h). This response was also accompanied by repression of genes associated with immune response and antioxidant activity.

Among other downregulated genes at 6 h, AT1G11670 and AT4G26690 play a role in cell wall organization and disease resistance. Together with genes associated with cell wall biosynthesis and cell expansion, like AT5G16910 (cellulose synthase-like D2), the general gene downregulation asset at 6 h reveals the involvement of cell wall-related components, which may possibly suggest a cell wall (re)organization upon treatment with self-DNA. Within the Energy, Metabolism, and Biosynthesis group (Table 1), downregulated genes like *gpat4*, *gpat5*, and *gpat7* are involved in lipid biosynthesis, and *pap3*, *pap27*, and *tps5* are involved in phosphate and trehalose metabolism, which are both essential for energy regulation and stress protection (Supplementary Dataset S4). Considering transport and signaling processes, AT2G25460 (C2 calcium-dependent membrane targeting); AT1G13210 (auto-inhibited Ca2+/ATPase II), involved in calcium ion transport; AT3G25610 (ATPase E1-E2 type family protein), which is a P4-type ATPase flippase that internalizes exogenous phospholipids across the plasma membrane; and AT5G24030 (SLAC1 homologue 3), involved in anion homeostasis, were all downregulated (Supplementary Dataset S4). Genes like ESL1 (AT1G08920), a sugar transporter across the vacuolar membrane, and PARG2 appear to be accompanied by the presence of genes encoding for transmembrane and vesicular transport and trafficking (AT3G14870, AT3G56200, AT1G17340, AT2G20960, AT5G41810, AT1G07000, and AT4G35380) or in signal transduction (AT1G08920, AT1G72540, AT1G18390, AT1G25390, AT2G41830, and AT4G04990) (Supplementary Dataset S4).

Upregulated genes at 6 h included stress responses associated with detoxification, revealed by two genes encoding peroxidases (AT4G08770 and AT5G64120) and a glutathione S-transferase (*gstu25*). In addition, genes associated with protein and nucleotide metabolism, encoding proteins involved in protein degradation (AT5G25340, coding for a ubiquitin-like superfamily protein), a prolyl oligopeptidase (AT1G20380), the uridine kinase-like 3 gene (AT1G55810), and the P-loop containing nucleoside triphosphate hydrolase (AT3G28580) were also upregulated. Two zinc transporters were upregulated at 6 h, and involvement of zinc transporters even expanded at 24 h. In parallel, upregulated genes related to growth and development were found, including the 2-oxoglutarate and Fe(II)-dependent oxygenase superfamily gene (AT1G12010) (Supplementary Dataset S4).

Downregulated genes at 24 h included HSP20-like chaperones (AT1G59860, AT1G07400, AT1G54050, AT5G12020, AT5G59720, AT4G10250, AT3G46230, AT1G53540, AT2G29500, AT5G52640, and AT1G16030) (Supplementary Dataset S4). The presence of the mitochondrion-localized small heat shock protein 23.6 (AT4G25200) may indicate that mitochondrial stress also possibly occurred. Members of the cysteine proteinase superfamily (AT5G50260 and AT3G48340) are also on the list, putatively involved in degrading damaged proteins, as well as genes encoding the glutathione S-transferase (GST) family (AT1G27140 and AT1G17190), known to be involved in detoxification, specifically removing reactive oxygen species (ROSs) during the stress response, and peroxidases (AT5G17820, AT4G30170, and AT5G39890), typically involved in ROS scavenging. A further set of downregulated genes at 24 h included AT5G17430, a key gene for embryo and somatic embryogenesis; AT5G10510, active in organ growth and development; the embryo defective genes (AT4G30090 and AT1G08840); and the root hair development-associated genes (AT5G67400 and AT1G27740), which are all genes associated with growth and development, along with genes associated with auxin response and hormonal signaling (AT4G12550 and AT5G17430) (Supplementary Dataset S4). Moreover, cellulose synthase-like and xyloglucan endotransglucosylase/hydrolase genes (AT3G56000, AT4G07960, AT4G30290, and AT5G23870), involved in cell wall biosynthesis and modification, and transporters (AT4G13420, AT5G40780) involved in the uptake of potassium and amino acids were downregulated at 24 h, as well as genes of the cytochrome P450 family (AT5G42580, AT4G37400, and AT3G01900) (Supplementary Dataset S4). Finally, we observed a downregulation at 24 h of ATPase and lipid metabolism (AT2G07698 and AT5G55050), and of *myb* and *nac* transcription factors (AT1G57560, AT3G15500, AT3G04070, and AT3G58120) (Supplementary Dataset S4).

Upregulated genes at 24 h are distributed in all 7 functional classes in Table 1 and Supplementary Dataset S4. Interestingly, among genes associated with the plant defense response, some encoding defensin-like (AT2G36255 and AT5G33355) and D-mannose-binding lectins (AT3G51710 and AT1G78820), together with the coronatine-induced protein (CORI3), which is part of the jasmonic acid signaling pathway, were found. Interestingly, we observed the presence of upregulated genes encoding chlorophyll A-B binding family protein (AT5G47110), typically involved in photosynthesis and light harvesting; nicotinamine synthase 2 (*nas2*), involved in iron homeostasis, crucial for photosynthesis; the iron-regulated transporter 3 (*irt3*), again supporting photosynthesis; and ferredoxin 1 (FD1), involved in electron transfer, especially in photosynthesis. Finally, some transcription factors were upregulated, such as basic helix-loop-helix proteins (*bhlh100*, *bhlh038*, and *bhlh039*) as well as *sco1*, a zinc finger transcription factor. Also, it can be noted that a total of 17 genes of unknown functions were differentially expressed, evenly distributed among upregulated and downregulated sets.

### 2.4. WGBS and Methylation Level in Treated vs. Control Samples

Genomic DNA was isolated from root tissue to perform a DNA methylation analysis at the single cytosine scale through bisulfite sequencing. On average, across the 15 samples, the extraction yielded 5 ng of purified DNA per mg of fresh-weight root with 260/280 and 260/280 ratios of 1.80 and 1.82, respectively, as measured by Nanodrop. WGBS library construction and DNA sequencing yielded an average of approximately 32 million sequence pairs per sample. Sequences were aligned to the reference genome with a mean mapping rate of 64% and converted into cytosine methylation levels. By requiring 10X as the minimum sequencing coverage for a reliable estimate of methylation levels, the analysis was restricted to approximately 2.2 million CG, 2.3 million CHG sites, and 11 million CHH sites per sample on average. Detailed information per sample is provided in Supplementary Table S2. The resulting dataset was used to evaluate the consistency of DNA methylation levels across sample replicates and time points by principal component analysis as well as identify differentially methylated regions between samples exposed to self-DNA and their controls.

**Figure 4:**
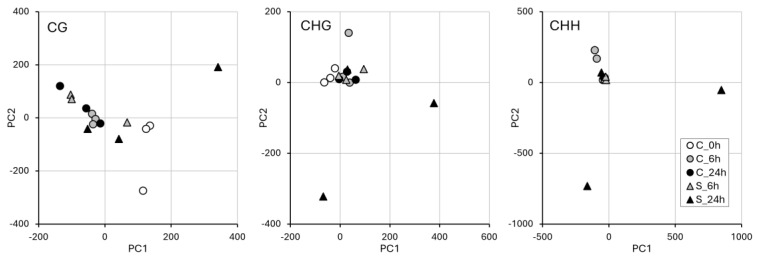
Results of PCA−based DNA methylation data. Panels refer to PCA for methylation contexts CG, CHG, and CHH, each showing experiment samples divided into clusters based on the variance explained by PC1 and PC2.

The exploratory PCA analysis (Figure 4) showed clear differences among the samples in the different methylation contexts (CG, CHG, and CHH). In particular, for the samples exposed to self-DNA for 24 h, there is an evident segregation of samples in the CHG and CHH contexts, whereas, in the CG context, most samples appear clustered, with only one self-treated sample resulting in separation along the first component, as in the other contexts. However, also in the control, an outlier sample was found in all the contexts. Distances among the three self-DNA-treated samples at 24 h could be related to overall read counts (Supplementary Table S2). In the other two methylation contexts (CHG and CHH), the sample spreading is similar, with a main cluster around the origin, a divergence of the outlier control sample (more evident in the CHG context), and a clear separation of two samples exposed to self-DNA for 24 h from the main cluster.

**Figure 5:**
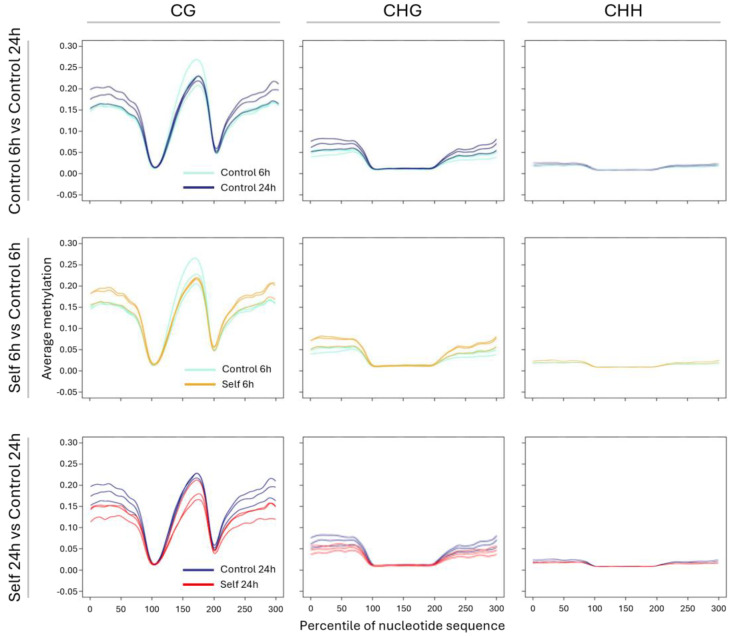
Results of meta−analysis for methylation levels in genic boundaries and intragenic regions. Data refer to the following comparisons: (**top**) controls at 6 h vs. controls at 24 h; (**center**) treatment with self−DNA at 6 h vs. controls at 6 h; (**bottom**) treatment with self−DNA at 24 h vs. controls at 24 h. Within each comparison (panel rows), data refer to the three explored contexts of methylation (panel columns from left to right: CG, CHG, and CHH). Within each plot, replicates within each treatment are symbolized with the same line color (see legend). X−axis values refer to the 2Kbp upstream gene boundary region (0−100), the intragenic region (100−200), and the downstream 2Kbp gene boundary region (200−300).

A meta-analysis of methylation levels in genic regions, specifically at the gene boundaries (upstream and downstream) and at intragenic levels (gene bodies), was carried out by binning cytosine methylation estimates across the length of genes (exonic sequence) and their 2 Kbp flanking regions. Figure 5 shows the results of this analysis for three fundamental comparisons, namely, between the controls at 6 h and 24 h, which monitors changes in DNA methylation during the time course that are not ascribed to self-DNA but only to the overall experimental setup, as well as between samples exposed to self-DNA and their respective controls at 6 h and at 24 h. DNA methylation profiles in all three types of comparisons, each showing the scenario for the CG, CHG, and CHH contexts separately, could be clearly interpreted in the upstream and downstream intergenic regions where between-replicates consistency was observed in contrast with the more unstable intragenic profiles exhibited by gene body methylation. The latter was expected to be restricted to the CG context, as only exonic regions, not directly affected by changes in methylation targeting transposable elements, were taken into account in this analysis. In the control time course, the variation in DNA methylation levels across the gene flanking regions, which are supposed to include both proximal gene regulatory regions, such as promoters, and repetitive elements linked to genes, seemed to have occurred in the transition between the 6 h and 24 h time points in the form of an average increase in methylation. Notably, relative to this control baseline, self-DNA exposure resulted in being accompanied by an increase in methylation already at 6 h. Nevertheless, during the time course, the roots exposed to self-DNA did not continue to maintain methylation levels higher than or at least equal to the control and instead returned at 24 h to the levels of methylation observed in controls at 6 h. The overall scenario could be described as a wave of DNA hypermethylation followed by a more intense demethylation in the roots treated with self-DNA despite an experimental setup that per se induced a general increase in methylation in the controls. This interpretation held true through a further analysis of methylation changes by significant DMR detection (Supplementary Figure S1), although the latter showed that the net hypermethylation and hypomethylation states shown by treated roots at 6 h and 24 h in the meta-analysis were actually the results of both gains and losses of methylation throughout the genome, with a prevalence of positive changes at 6 h and negative changes at 24 h relative to the control baseline (Wilcoxon rank sum test, *p*-value < 2 × 10^−16^).

### 2.5. Association Between DMR Occurrence and Differential Gene Expression

DNA methylation meta-analysis revealed that the major discrepancies in methylation levels between control and treated roots mainly resided in the intergenic region and were progressively less ample in magnitude while approaching the genes’ boundaries. This notwithstanding, we set out to quantify more precisely the impact of DMRs on the gene space, including in this definition both gene bodies and 1 Kbp proximal promoters. Significant DMRs present in gene bodies of differentially expressed genes amounted to 79, distributed among 61 genes (Supplementary Dataset S5). Sixty-three of these DMRs involved CHH sites, while in 12 DEGs, both CG and CHH DMRs were detected (Supplementary Dataset S5). Methylation differences between treated and control roots in these regions rarely exceeded four units on a scale of 100, and the overall correlation between methylation and gene expression variation was poor (R^2^ = 0.0054), thereby making their functional significance elusive under the assumption, likely too conservative, of a unique regulatory model for all these genic regions. Nevertheless, one case stood out for the presence of a series of combined CG/CHG/CHH DMRs within an interval of 200 bp in the coding sequence of the AT2G07698 gene, which codifies for the F1 portion of the ATP synthase complex. With a mean difference of approximately −10 percentage units of CG and CHG methylation between treated and control roots at 24 h, this represented a robust case of hypomethylation occurring during exposure to self-DNA treatment at 24 h, which corresponded to a downregulation of the gene expression level.

Intersecting DMRs with promoters of DEGs revealed only 39 matches (Supplementary Dataset S6), nearly all of which involved CHH DMRs. Here too, a single DEG gene raised interest for a rare CG DMR in its promoter and a hypomethylated condition (−25 percent methylation units) at 24 h when the gene AT4G07960, predicted to encode an XyG glucan synthase, a crucial polysaccharide in plant cell wall structure and function, particularly for cell growth and development, was strongly downregulated.

## 3. Discussion

The inhibitory role of fragmented, extracellular self-DNA has been reported in several organisms [41,42,43,44,45,46]. According to previous reports, self-DNA may also function as a stress-signaling molecule (DAMP) [37]. Upon cell and tissue injury, DAMPs are released, inducing a rapid response via PRRs involving single ions like calcium, reactive oxygen species, and mitogen-activated protein kinases [36,37]. Responses to biotic and abiotic stress in model plants have been recently reviewed (e.g., in [47]), and a metabolomics and molecular characterization of differential responses to self-DNA as compared to other stressors has also been pointed out [48]. An increased cytoplasmic content of cAMP, cGMP, and N6-methyl-AMP as an exclusive response to self-DNA was interpreted as an accumulation of RNA constituents consistent with a reduced transcription activity as resulting from gene expression evidence [37]. In the context of gene expression, exposure to self-DNA has been shown to induce differential activation of genes known to be responsive to abiotic stress, such as heat, desiccation, and oxidative stress, as well as defense genes [34,35,38,39,40]. Early responses to these stress signals (up to a few hours from exposure start) have been covered by Duran-Flores and Heil [37] and Barbero et al. [36]. However, information on later molecular responses and how exogenous self-DNA can influence gene expression remains unclear.

In this study, we presented the first exploratory results of RNA-seq and WGBS data from a manipulative case-control experiment on *Arabidopsis thaliana* roots exposed to self-DNA, built on an original experimental setup to ensure exposure was limited to the roots of the plant seedlings. The complex experimental setup we designed was fundamental to investigating the changes in gene expression levels, although partially disturbed by variability within treatments and cytosine methylation patterns between samples treated with self-DNA and control at 6 and 24 h of exposure, including an association study between DMR occurrence and differential gene expression. In general, our results showed clear trends in the two time points for what concerns gene expression, accompanied by changes in DNA methylation patterns that provide the first overall picture of the effects of exposure to self-DNA in roots. The mechanisms regulating plant root response to exposure to self-DNA at cellular and physiological levels cannot be fully elucidated in the absence of additional data on, for example, proteomics and metabolomics, which can provide milestones along the long way that starts from gene expression. However, some of the basic effects, also considering previous evidence on the same species at gene expression on the whole plant [43] and at the metabolomics level [48], are highlighted in the following text.

### 3.1. Differentially Expressed Genes at 6 h Exposure

The analysis of gene expression at 6 h and 24 h in roots, while confirming the overall picture already detected in Arabidopsis plant seedlings when comparing the gene expression patterns determined by the exposure to self-DNA and nonself-DNA [43], nonetheless reveals additional interesting aspects along the experimental time course.

Downregulated genes at 6 h are associated with stress and defense response, cell wall biosynthesis, signal transduction, and ion transport, as well as with metabolic processes. In the case of genes associated with the stress and defense response, the observed pattern may suggest a release of the plant functions possibly active during the preceding stages, before 6 h, after stress solving or acclimation. Indeed, the repression of pathogen-responsive genes like RPS6 (AT5G46470) suggests, as previously proposed [36,37], that the exposure to self-DNA activates an early priming of the plant immune response (before 6 h) in analogy to what is exerted by exposure to pathogens. Interestingly, genes associated with the response to *P. siringae* were also observed in seedlings exposed to self-DNA [43].

Genes involved in protein folding and maintenance, especially under stress conditions, were downregulated at 6 h, possibly suggesting the occurrence of stress-induced protein misfolding in plant roots, possibly related to the stressing effect of treatment that can elicit abiotic stress response [39]. Differential expression of some genes associated with growth regulation, like AT4G26690, a glycerophosphoryl diester phosphodiesterase-like protein involved in cell wall cellulose accumulation and pectin linking, with others associated with cell wall biosynthesis and cell expansion, like AT5G16910 (cellulose synthase), AT4G30270 (xyloglucan endotransglucosylase/hydrolase), and AT4G39400 (brassinosteroid insensitive), all related to the remodeling of the cell wall, may indicate avoidance processes triggered by self-DNA exposure [43].

Genes that can be associated with downregulation of cell division and growth (like separase homolog, cellulose synthesis, and exocytosis), together with ion transport and signaling, may support the interpretation of some cellular response mechanisms previously proposed from self-DNA exposure in plants [36,43,48] and other organisms [44,45], among which are the inhibition of cell proliferation and alteration of cell cycle, as assessed by cytofluorometry in yeast [46,49].

The analysis of upregulated genes at 6 h reflects a balance of genes involved in stress responses, metabolism, transport, development, and cellular maintenance in the plant. Some of these genes are associated with oxidative stress management, which may reflect the reaction to ROS production after exposure to self-DNA [43], while other genes appear to be critical for developmental processes such as those associated with root differentiation. In addition, the two zinc transporters upregulated at 6 h, and their larger numbers at 24 h, may also indicate an increased demand for or redistribution of zinc within the root system [43]. Since zinc plays a role in many enzymes and cellular functions, higher levels of zinc in the root could highlight increased metabolic activity initiating at 6 h and increasing at 24 h, also associated with detoxification processes, or be associated with zinc requirements because of a more extensive consumption. This is also supported by the upregulation of a gene belonging to the Major Facilitator Superfamily (AT1G68570). This gene could be involved in transporting a variety of molecules across the root cell membranes, such as sugars, amino acids, or other essential metabolites, being crucial for nutrient uptake, ion balance, and growth processes. Therefore, its upregulation suggests increasing molecular transport activity in the root.

The increased expression of detoxification and oxidative stress-related genes (e.g., peroxidases, GSTs) at 6 h may suggest the root is still experiencing stress. The upregulation of genes involved in lateral root development (e.g., *lbd41*) suggests active regulation of root architecture, which could be linked to self-DAN avoidance. The presence of transcription factors and enzymes involved in signaling is consistent with a molecular reprogramming of roots in response to their environment, which may involve adjusting growth, metabolism, and stress responses. Overall, the upregulation of these genes points to a root system that is actively restarting adaptation to stresses while promoting growth, nutrient acquisition, and possibly mechanisms associated with growth and development.

### 3.2. Differentially Expressed Genes at 24 h Exposure

The pattern of gene downregulation at 24 h is consistent with a release of the response to stress, including responses associated with protein folding, stress signaling, and root and development. Speculatively, root growth-related processes (such as root hair development) at this stage may be relaxing in the presence of a second phase of stress response, where recovery and reorganization are occurring, while detoxification is still needed.

At 24 h, the downregulation of additional HSPs when compared to those repressed at 6 h, also including HSPs related to mitochondrial stress, highlights a modulation of HSP production in plant root stress response accompanying changing conditions during the experimental time course. Indeed, while some genes are exclusively downregulated at 6 h, at 24 h, the plant stress response involves additional genes, possibly associated with a later response, while its action towards detoxification activities, including ROS removal, is still ongoing. The low number of genes upregulated at 6 h becomes much higher at 24 h. Among these, defensins, lectins, and resistance proteins suggest that at this stage, the seedling root is still under stress conditions, which may be a secondary effect of the exposure to self-DNA given the absence of pathogens from the experimental system. The upregulation of direct (like *sep2*, *var2*, and *gun5*) or indirect (like *ndf1*, *ndf2*, ftsh1, lhcb5, and *sige*) ROS-related genes indicates that oxidative stress is still in effect, consistent with previous studies [43]. At this stage, upregulation of glutathione S-transferases may correspond to ROS scavenging, indicating that the detoxification action in plant roots is ongoing. Accordingly, upregulation of defensive proteins such as *defl* (lectins), known for a defensive role against pathogens, would suggest that self-DNA exposure can produce effects similar to those triggered by pathogen attack, such as ROS production. In support, coronatine-induced protein (*cori3*), which is part of the jasmonic acid signaling pathway activated under stress, particularly by pathogens, and specifically by wound response, may be related to the sensing of self-DNA being detected as a damage-associated molecular pattern (DAMP, [34]). Accordingly, the upregulation of genes associated with photosynthesis and iron homeostasis, involved in direct or indirect ROS management (chlorophyll A-B binding family protein (AT5G47110), *nas2*, and *fd1*), and additional transporters (*mrp*, AT5G13580, and *zips*) would correspond to a protective action against oxidative stress, accompanied by membrane and protein turnover to address damage while reactivating energy-requiring metabolic processes. This would be consistent with the upregulation of some genes involved in protein and lipid metabolism, such as *ftsh1* and prolyl oligopeptidase (AT1G20380), phospholipase-like protein (*pearli4*), alpha/beta-hydrolases superfamily protein (AT4G36610), 3-ketoacyl-CoA synthase 3 (*kcs3*), acyl-activating enzyme 18 (*aae18*), and fructose-bisphosphate aldolase 1 (*fba1*), with a key role in sugar metabolism and, particularly, in glycolysis.

Finally, the upregulation of S-adenosyl-l-homocysteine hydrolase 2 (*sahh2*), which is involved in methylation reactions and metabolic regulation, could be associated with cellular methylation processes, which may impact gene expression, in possible association with the DNA methylation results.

### 3.3. Epigenetic Response to Self-DNA Exposure

We here present the first WGBS associated with RNA-seq data from a manipulative case-control experiment on *Arabidopsis thaliana* roots exposed to self-DNA. The only preceding study presenting WGBS data in plants exposed to self-DNA [34] targeted *Lactuca sativa* seedlings and only the CG methylation context, showing no changes in DNA methylation at inhibitory self-DNA dosage (200 mg mL^−1^) and a slight decrease in methylation percentage at lower dosages (from 2% at 2 mg mL^−1^ to 3% at 150 mg mL^−1^).

Our results, based on an original experimental setup to ensure and limit self-DNA exposure to seedling roots, resulting in a low dose per plant (200 µg DNA in 1 mL of treatment solution shared by several seedlings (see the Section 5)), showed a change in seedling root DNA methylation profiling in all methylation contexts, with a different pattern according to exposure time. The results associated with the three methylation contexts, and, in particular, the comparison between the unexposed controls at 24 h vs. 6 h, showing an increased methylation at the flanking gene regions, indicate that our original experimental setup was somehow stressful for the seedling roots, under the established assumption that methylation is associated with exposure to environmental stress [50]. Similarly, exposure to self-DNA at 6 h induces an increased methylation at the flanking gene regions, while after 24 h of exposure, a drastic reduction in the levels of methylation was observed at the flanking gene regions in all three contexts, the scenario being less clear within gene bodies, where CG methylation is prevalent.

DNA methylation in *A. thaliana* is a pivotal epigenetic mechanism that is involved in two processes: protecting the genome from selfish DNA elements (e.g., transposons) and regulating gene expression [51]. Recently, it has also been reported that DNA methylation plays a role in regulating genome functioning to induce plant resistance and adaptation to abiotic stresses (drought, high temperature, cold, salt, and heavy metal stresses) [50]. In our study, the variation in DNA methylation levels may indicate a stress response mechanism associated with DNA epigenetic reprogramming. The variability of the methylation asset at the two different time points can be discussed in association with the stress response waves detected by the differentially expressed genes (DEGs) analysis, indicating a relationship between DNA methylation and gene expression patterns.

Indeed, the DNA methylation pattern revealed at 6 h, indicative of an increased methylation at intergenic regions, may putatively be associated with the first wave of the stress response. We could not provide any evidence for a parallel massive change in gene expression that could be promoted by DMRs, as only a few of these co-mapped with genes and gene promoters. Also, we could not detect genes that could actively play a role in this first genetic reorganization, and this is probably related to the timing of the sampling stage. However, it is reasonable to consider that the increased DNA methylation in intergenic regions could be a direct consequence of an earlier, massive dysregulation of gene expression as previously observed in response to self-DNA exposure [43], although the prevailing gene downregulation observed in [43] as a result of self-DNA exposure and the massive hypermethylation observed at 6 h in the present study cannot be easily reconciled in this reverse order. Inferences drawn from the combination of the two studies warrant particular attention, as it should also be noted that they were conducted in different experimental conditions, i.e., at higher self-DNA concentration and on the whole-plant transcriptome in that previous study and at lower DNA concentration and on the root transcriptome in the present study. Nevertheless, the molecular stresses experienced by the plants exposed to self-DNA are a common theme that could guide the interpretation of another distinct signature emerging from the current experiment, that is, the second wave of DNA methylation resetting, in the form of hypomethylation, which occurs at 24 h and strikes for the relative precocity of this rebound compared to the initial hypermethylation. Such massive epigenetic changes are reminiscent of critical events linked to reproduction and early embryo development in conditions that are completely different from our experimental conditions.

Among the DEGs apparently most affected by DNA hypomethylation, two remarkably downregulated genes somehow suggested a consistent pattern with respect to previous observations from physiological and metabolomics studies. In particular, the massive downregulation of AT2G07698, which codifies for the F1 portion of the ATP synthase complex, would correspond to a decrease in ATP and then to a possible accumulation of its precursor nucleosides, consistent with the metabolomics evidence and interpretation by Lanzotti et al. [48] who recently showed also the reversibility of the self-DNA inhibitory effects after exposure to nonself-DNA [52]. In addition, the downregulation associated with hypomethylation of AT4G07960, encoding a xyloglucan synthase, might indicate an alteration or rearrangement of root cell walls, somehow consistent with a response of physical avoidance of self-DNA fragments (which, according to [43], would persist at apoplastic location during root exposure), including the development of adventitious rootlets and root hairs, previously observed at low self-DNA concentration [43].

However, the overall epigenetic pattern emerging for the present study can hardly be supportive of the mechanism of self-DNA acting as a DAMP, which has gained much attention in the last years [34,35,37,40]. Indeed, we failed to find differential DNA methylation in treatment vs. control comparisons related to plant immunity and resistance genes. However, this may be related to the low dose per plant characterizing our experimental setup as compared to previous studies. It might also be possible that DNA methylation changes induce mechanisms to control gene expression under these stress conditions. DNA is an essential molecule for organisms that contains the information for survival. As a consequence of evolution, cells are able to detect several pathogen-derived or host-derived substances released when there is damage, including DNA [53]. DNA recognition by the organism is necessary for the aforementioned process to occur. Recognition of self-DNA and nonself-DNA is the role of the Pattern Recognition Receptors (PRRs) [54]. In mammals, DNA recognition is carried out by Toll-like receptor (TLR9), cyclic GMP–AMP synthase, and “absent in melanoma 2” (AIM2), depending on its localization in either the endosomal compartment or the cytoplasm [54]. The mechanism of DNA recognition by plant cells or the function of extracellular self-DNA in the organisms is still unclear [41,42]. Although plants have putative PRRs, no extracellular DNA receptor has been identified, but PRRs are proposed as good candidates for these receptors [54]. Some authors consider that the recognition of DNA in plants is similar to that presented by animals via sensors, including TLR9 [55].

## 4. Conclusions

Our study, based on an original experimental setup to ensure and limit exposure to self-DNA and transcriptome and DNA methylome analyses to seedling roots, confirmed and contributed further evidence concerning gene expression response to exposure to self-DNA in *Arabidopsis* roots. The observed pattern of downregulation of stress-responsive genes suggests main waves in the response to self-DNA: the first wave, representing the initial stress response caused by the treatment, associated with systemic signaling, mediated by calcium and ROS production, inhibiting membrane trafficking and therefore transmembrane transport, should have occurred before 6 h after treatment. This may cause nutrient deprivation, hypoxia-related effects, and energy requirements, determining growth inhibition. At 6 h, the plant appears to enhance basic activities like membrane transport and signaling, putatively recovering from self-DNA inhibitory effects. At 24 h, gene expression analysis reveals activation towards cell component turnovers, possibly to recover from damages, while detoxifying and reorganizing the overall structural asset. Upregulation of genes related to cell wall modification and calcium signaling suggests that the root is experiencing active growth, expansion, and possibly adaptation to the new conditions, which may be related to self-DNA physical avoidance. The evidence that several genes remain uncharacterized at both time points indicates the potential for further functional exploration of the response to self-DNA. The corresponding epigenetic pathway, here shown for the first time in seedling roots and for all three methylation contexts, might be a possible mechanism in DAMP signaling. However, DNA methylation changes could induce mechanisms to control gene expression under the stress of self-DNA exposure, as we showed for root cell wall reorganization and accumulation of RNA degradation byproducts. Though identification of DNA methylation regions is critical and fundamental to identifying the functional regions that may be involved in transcriptional regulation, our explorative analysis provided valuable insights on the molecular response to self-DNA. Also, we tested an innovative experiment setup that will help future investigations on the topic that could address whether the metabolomics reversibility of self-DNA inhibition has a corresponding plasticity in the epigenetic responses.

## 5. Materials and Methods

### 5.1. Seed Sterilization and Germination

**Figure 6:**
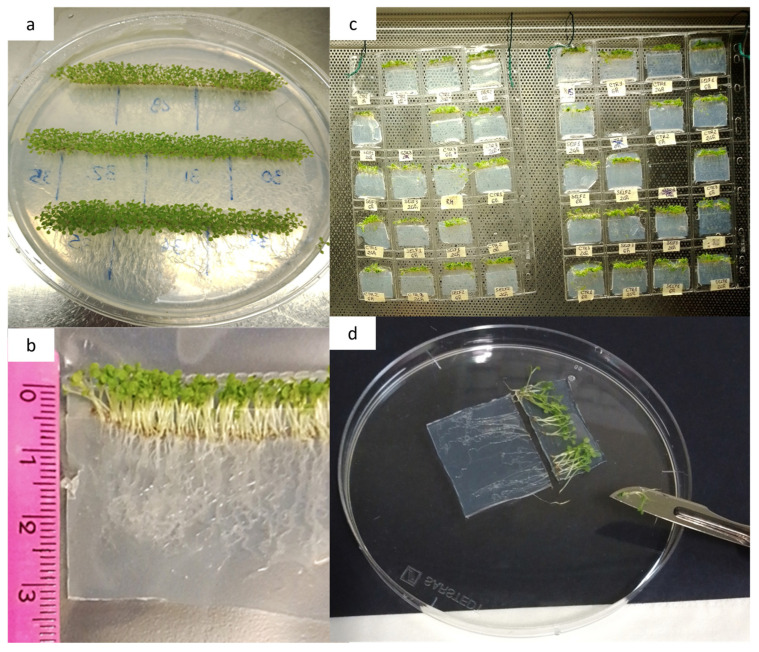
Experimental preparation of slices for root exposure to self-DNA. (**a**) Growth medium containing seedlings divided into slices by marking and measuring designated cuts on the back of a Petri dish using a marker and ruler. (**b**) Demarcated slices (~35 mm × 40 mm) were carefully cut using a sterile scalpel within a sterile hood environment. (**c**) Each slice was carefully placed inside a pocket of a 20-pocket plastic coin holder sheet (pocket size 45 mm × 30 mm). The roots were completely enclosed within the plastic pocket, while the green top portion (0.5–1 cm) remained outside of it. Once filled with the slices, the plastic coin holder sheets were hung under a sterile hood and kept as straight and firm as possible. (**d**) Collected slices were gently put on a Petri dish lid, and the green top was cut off with a sterile scalpel.

Sterilized seeds (1% NaClO for 5 min., then rinsed 5× for 1 min. with sterile water) of *Arabidopsis thaliana* Col 0 were distributed on fifteen 150 mm Petri dishes (i.e., replicates) in lines using a pipette. Dishes were filled with a thin layer of Murashige and Skoog growth medium (1 L made with 4.4 g Murashige–Skoog basal medium with Gamborg’s vitamins—Sigma Aldrich (Burlington, MA, USA); 30 g sucrose, 16 g agar, and sterile water to final volume). Once hermetically closed, dishes were placed in a growth chamber under controlled environmental conditions (22 ± 2 °C, 50% RH, 16/8 h day/night photoperiod) for 15 days, leaning on an inclined plane to favor geotropism of root growth and prevent root spreading inside the growth medium (Figure 6a).

### 5.2. Growth Medium Slices and Control Samples Preparation

Once the seedling roots reached approximately 3 cm in length, the growth medium with the seedlings was sectioned into 35 mm (length) × 40 mm (height) slices using a sterile scalpel under a sterile hood. A total of 60 slices, selected for having the highest density of Arabidopsis seedlings (Figure 6b), were chosen for the experiment. Each slice was carefully placed into a pocket of a 20-pocket plastic coin holder sheet (each pocket measuring 45 mm × 30 mm). The 3 cm long roots were fully enclosed within the plastic pocket, while the green shoot portion, approximately 0.5–1 cm, remained outside. The three plastic coin holder sheets, each filled with slices, were suspended under a sterile hood to maintain their shape and orientation as straight and stable as possible (Figure 6c).

### 5.3. Sample Collection and Preparation

Each biological replicate consisted of four randomly selected tissue slices. To establish baseline controls (time 0 h), twelve slices were immediately collected and grouped into three unexposed control replicates. The slices were placed gently onto a Petri dish lid, where the green shoot was removed using a sterile scalpel under a laminar flow hood. For each replicate (i.e., each set of four slices), roots were carefully retrieved with a sterile spatula (Figure 6d), rinsed with sterile water, dried on paper, and weighed. The root material from each replicate was split into two Eppendorf tubes: one containing approximately 50–70 mg for DNA extraction and the other 15–25 mg for RNA extraction. All tubes were labeled accordingly and stored at −80 °C for future analysis.

### 5.4. Self-DNA Solution Preparation

Self-DNA solution was prepared using DNA extracted from fresh A. thaliana leaves. The process included a quality check, quantification, RNase A treatment, and sonication, following established protocols [37]. Leaf tissue (1 g) was ground in liquid nitrogen and mixed with CTAB buffer (0.1 M Tris-HCl pH 7.5, 0.7 M NaCl, 0.01 M EDTA, 1% CTAB). The mixture was incubated at 65 °C for 45 min with intermittent mixing. An equal volume of chloroform/isoamyl alcohol (24:1) was added, followed by centrifugation at 14,000× *g* for 20 min. The aqueous phase was transferred to a new tube and precipitated with an equal volume of isopropanol at −20 °C for at least one hour. DNA pellets were recovered by centrifugation at 4 °C, washed with cold 70% ethanol, air-dried, and resuspended in 200 µL of sterile water. RNase A (0.25 mg/mL) was added and incubated at 37 °C for 1 h, then inactivated at 70 °C for 20 min. Two technical replicates were prepared per sample. DNA quality was assessed by Nanodrop ND-1000 (Thermo Fisher, Waltham, MA, USA), and concentration was measured using a Qubit 3.0 fluorometer (Life Technology, Carlsbad, CA, USA). Gel electrophoresis (1% agarose) was used to confirm DNA integrity. Sonication was performed using a Bioruptor Plus (Diagenode, Liege, Belgium) for 12 min (60 s ON/30 s OFF) to produce fragments between 200 and 1000 bp, a range previously shown to be most inhibitory to root elongation in vitro [35,37] (Supplementary Figure S2).

### 5.5. Root Exposure and Sampling

Following initial control sample collection (Section 5.2), 24 pockets containing tissue slices were treated with 1 mL of the prepared self-DNA solution (~60 ng/µL), while others received 1 mL of sterile water. During the exposure, airflow within the hood was maintained, and lights were turned off to minimize temperature increase and evapotranspiration. After 6 h of exposure, 24 additional slices were harvested—12 exposed to self-DNA (3 replicates of 4 slices each) and 12 treated with sterile water (3 replicates). Root processing followed the same method as earlier: washing, separation into DNA and RNA aliquots, and storage at −80 °C. This procedure was repeated 24 h after the beginning of the exposure. In total, 15 samples were collected: 3 untreated controls at 0 h, 3 controls and 3 DNA-treated at 6 h, and 3 controls and 3 DNA-treated at 24 h. As each sample yielded two tubes (DNA and RNA), a total of 30 Eppendorf tubes were properly labeled and stored for analysis.

### 5.6. RNA Extraction and mRNA Sequencing

RNA was extracted from each of the 15 samples using the tube containing ~15–25 mg of root tissue. The Spectrum™ Plant Total RNA Kit (Sigma-Aldrich, Burlington, MA, USA) was used, with reagent volumes adjusted for small sample size: 300 µL of Lysis Solution/2-ME mixture, 500 µL of Binding Solution, 300 µL per wash step, and a single 35 µL elution. RNA quality was evaluated with a Nanodrop ND-1000 spectrophotometer and 1% agarose gel electrophoresis. Concentration was measured using the Qubit RNA Broad-Range Assay Kit and Qubit 3.0 fluorometer (Life Technology, Carlsbad, CA, USA). RNA integrity was determined using an Agilent 2100 Bioanalyzer at IGA Technology Services (IGATech, Udine, Italy). mRNA libraries were prepared using the Universal Plus mRNA-Seq Kit (Tecan Genomics, Männedorf, Switzerland), following manufacturer instructions (fr-secondstrand library type). Final libraries were verified using both a Qubit 2.0 fluorometer and an Agilent Bioanalyzer DNA assay. Libraries were sequenced using paired-end 150 bp reads (~23 million spots per sample, totaling 7 Gbp) on a NovaSeq 6000 platform (Illumina, San Diego, CA, USA), with library prep and sequencing conducted by IGATech.

### 5.7. DNA Extraction and Whole Genome Bisulfite Sequencing (WGBS)

DNA was extracted from the tubes containing ~50–70 mg of root tissue using the MagMAX Plant DNA Isolation Kit (Thermo Fisher Scientific, A32549, Waltham, MA, USA), following the manufacturer’s protocol. DNA purity and quantity were assessed using a Nanodrop ND-1000 spectrophotometer and Qubit 3.0 fluorometer with the Qubit Broad-Range DNA Assay Kit. At least 100 ng of DNA per sample was used for WGBS. Bisulfite conversion, library preparation, and sequencing were conducted at IGATech using the Ultralow Methyl-Seq System (Tecan/NuGEN, Zürich, Switzerland), which generates directional libraries. Forward reads correspond to bisulfite-converted versions of the original strands (C-to-T), while reverse reads represent their complements (G-to-A). Final library quality was checked using the Qubit 2.0 fluorometer and Agilent Bioanalyzer DNA assay. Sequencing was performed in paired-end 150 bp mode (~23 million paired reads per sample) on a NovaSeq 6000 platform.

### 5.8. Bioinformatics Analysis

Raw reads from both RNA-seq and WGBS were processed with TrimGalore [56] for adapter trimming and low-quality base removal and quality-checked with FastQC [57]. WGBS reads were deduplicated to eliminate PCR artifacts, retaining only unique start and end positions [58]. Mapping to the *Arabidopsis thaliana* TAIR10 reference genome and conversion to cytosine-specific methylation profiles were performed using Bismark v0.22.3 [59]. DNA methylation-based PCA and DMR detection were performed using methylKit in R [60], with default settings and a Q-value cutoff of 0.01.

RNA-seq reads were aligned to the TAIR10 genome using STAR v2.7.9a [61], with gene-level counts generated using the “-quantMode GeneCounts” option. Principal component analysis (PCA) was performed on normalized read counts (regularized-logarithm transformation, DESeq2 [62]) and visualized with ggplot2 [63]. Differentially expressed genes (DEGs) were also identified using DESeq2.

All biological replicates (n = 3) per experimental group—defined by treatment (self-DNA or control) and time point (0 h, 6 h, or 24 h)—were included in PCA plots for both WGBS and RNA-seq datasets to assess replicate variability. Visual inspection of PCA plots was used to identify outliers, which were subsequently excluded from further analysis.

## Figures and Tables

**Table 1:** Groups of DEGs according to their functions. Data refer to counts of downregulated (blue header background) and upregulated (red header background) DEGs in the comparisons of control vs. exposed to self-DNA at 6 h. and 24 h. Counts of DEGs consistently downregulated or upregulated in both comparisons are also reported in the (6 and 24 h) columns.

Group of DEGs	Down				Up		
	6 h	6 and 24 h	24 h		6 h	6 and 24 h	24 h
***1. Stress and Defense***							
Stress Response/Defense: Detoxification/ROS	-	-	2		1	-	-
Stress Response/Defense: Others	-	-	2				
Stress Response: Cysteine Proteinases	-	-	2				
Stress Response/Defense: Detoxification/Glutathione	-	-	2				
Early Response to Dehydration	1	-	-				
Stress Response and Protein Quality Control	4	1	-				
Heat Shock Proteins	7	5	-				
Defense and Pathogen Response	4	1	-				
Reactive Oxygen Species (ROSs) Response and Detoxification					-	-	3
Detoxification					3	-	-
Direct ROS Management					-	-	5
Indirect ROS Management					-	-	4
Defense Proteins					-	-	7
***2. DNA/Protein Processing and Folding***							
Protein Modification and Degradation					3	1	-
Nucleotide Metabolism					2	-	-
Protein Folding and Degradation					1	1	4
***3. Growth and Development***							
Cell Wall and Growth Regulation	6	-	-		4	1	-
Embryo Development and Regulation	-	-	4				
Root Hair Development	-	-	2				
Cell Wall Synthesis and Modification	-	-	4				
Cell Cycle and Division (Cyclins)	-	-	1				
Embryo and Seed Development					-	1	4
Chlorophyll/Photosynthesis-related					-	-	1
Cell Wall-Modifying Enzymes					-	-	2
***4. Transport, Signaling, and Homeostasis***							
Ion Transport and Signaling	5	-	-		2	3	-
Signal Transduction and Membrane Functions	5	1	-				
Transport and Membrane Trafficking	6	1	-				
Cellular Homeostasis and Protection	3	-	-				
Nutrient and Ion Transport	-	-	3				
Hormone and Signaling: Auxin Signaling	-	-	1				
Hormone and Signaling: Auxin and Cytokinin Signaling	-	-	1				
Hormone and Signaling: Terpenoid Synthesis	-	-	1				
Cytochrome P450s	-	-	3				
***5. Energy, Metabolism, and Biosynthesis***							
Metabolism and Biosynthesis	9	2	-		-	-	9
DNA/RNA Processing	-	-	1				
ATP Production and Regulation	-	1	1				
Nucleotide Metabolism					2	-	-
Redox Balance and Electron Transport					-	-	4
Metabolism and Transport					-	2	9
Lipid Metabolism (Acyl-CoA)					-	-	8
Sugar Metabolism and Transport					-	-	4
**6. Gene Expression and Transcription Regulation**							
Transcription Factors (TFs)	-	-	1		-	-	7
NAC Domain TFs	-	-	5				
**7. Uncharacterized/Unknown Proteins/Miscellaneous Roles**	2	-	8		2	1	4

## Data Availability

All relevant data are included in the manuscript and Supplementary Materials.

## Supplementary Materials

The following supporting information can be downloaded at: https://www.mdpi.com/article/10.3390/epigenomes9020013/s1, Supplementary Datasets S1: DEGs at 6 h; Dataset S2: DEGs at 24 h; Dataset S3: GO enrichment; Dataset S4: DEGs by function; Dataset S5: Association between DMR and DEGs in gene bodies; Dataset S6: Association between DMR and DEGs in gene promoter regions. Supplementary Table S1: Sequencing statistics of transcriptomic data; Supplementary Table S2: Sequencing statistics of WGBS data. Supplementary Figure S1: Histograms of DMR from treatment vs. control comparisons for all time points and methylation contexts; Supplementary Figure S2: Agarose gel electrophoresis of self-DNA samples used for treatment solution preparation.
