# Supplementary material for: Arabidopsis thaliana Roots Exposed to Extracellular Self-DNA: Evidence of Epigenetic Effects
Source: Epigenomes. 2025 Apr 30;9(2):13. doi: 10.3390/epigenomes9020013 (PMC12101399; doi:10.3390/epigenomes9020013)

*Arabidopsis thaliana* Roots Exposed to Extracellular Self-DNA: Evidence of Epigenetic Effects

Alessia Ronchi ^1,2^, Guido Incerti ^1^*, Emanuele De Paoli ^1^, Speranza Claudia Panico ^1^, Giovanni Luca Sciabbarrasi ^1^, Pasquale Termolino ^3^, Fabrizio Cartenì ^4^, Mariachiara Langella ^4^, Chiusano Maria Luisa ^4^ and Stefano Mazzoleni ^4^*

^1^ University of Udine, DI4A, via delle Scienze 206, 33100 Udine, Italy

^2^ University of Padova, DAFNAE, via dell’Università 16, 35020 Legnaro (PD), Italy

^3^ National Research Council, Institute of Biosciences and Bioresources, 80055 Portici (NA), Italy

^4^ University of Napoli Federico II, Dipartimento di Agraria, via dell’Università 100, 80055 Portici (NA), Italy

***** Correspondence: [guido.incerti@uniud.it](mailto:guido.incerti@uniud.it); [stefano.mazzoleni@unina.it](mailto:stefano.mazzoleni@unina.it)

**SUPPORTING INFORMATION: Supplementary Tables and Figures**

**Supplementary Table S1.** **Sequencing statistics** **of transcriptomic data**. List of IDs, descriptions, sequencing depth, quality and mapping produced for each RNA sample.

| **Sample_ID** | **description** | **Sequence pairs** | **Unique best hits** | **Mapping efficiency (%)** |
| --- | --- | --- | --- | --- |
| CTRL1 | RNA CTR1 0h | 20559689 | 17270139 | 84.0% |
| CTRL2 | RNA CTR2 0h | 22610638 | 19354706 | 85.6% |
| CTRL3 | RNA CTR3 0h | 19261261 | 16564684 | 86.0% |
| CTRL4 | RNA CTR4 6h | 26303339 | 24041252 | 91.4% |
| CTRL5 | RNA CTR5 6h | 14775153 | 12100850 | 81.9% |
| CTRL6 | RNA CTR6 6h | 18776424 | 16523253 | 88.0% |
| CTRL7 | RNA CTR7 24h | 23951778 | 21173372 | 88.4% |
| CTRL8 | RNA CTR8 24h | 52925608 | 42605114 | 80.5% |
| CTRL9 | RNA CTR9 24h | 19877313 | 16597556 | 83.5% |
| SELF1 | RNA SELF1 6h | 27936420 | 22600564 | 80.9% |
| SELF2 | RNA SELF2 6h | 44827996 | 40255540 | 89.8% |
| SELF3 | RNA SELF3 6h | 41081897 | 36973707 | 90.0% |
| SELF4 | RNA SELF4 24h | 25934300 | 23263067 | 89.7% |
| SELF5 | RNA SELF5 24h | 22087689 | 13208438 | 59.8% |
| SELF6 | RNA SELF6 24h | 29102946 | 25814313 | 88.7% |

**Supplementary Table S2. Sequencing statistics of WGBS data**. List of IDs, sequencing depth, quality, mapping and coverage (number of position and mean percent coverage) for the three methylation context in the final dataset.

| **Sample ID** | **Sequence pairs** | **Unique best hits** | **Mapping efficiency (%)** | **Minimum coverage  (all pos)** | **Final Dataset** | | | | | |
| --- | --- | --- | --- | --- | --- | --- | --- | --- | --- | --- |
|  |  |  |  |  | **CG  positions** | **Mean coverage** | **CHG positions** | **Mean coverage** | **CHH positions** | **Mean coverage** |
| CTRL1 | 31529796 | 20806692 | 66.0 | 10 | 2049824 | 25.2 | 2122442 | 22.6 | 10538241 | 21.3 |
| CTRL2 | 29341162 | 20091362 | 68.5 | 10 | 2431956 | 23.3 | 2511333 | 21.0 | 12347285 | 19.9 |
| CTRL3 | 26689250 | 17901264 | 67.1 | 10 | 2054824 | 23.2 | 2108611 | 20.8 | 10353238 | 19.6 |
| CTRL4 | 38048196 | 27986405 | 73.6 | 10 | 4527147 | 17.3 | 4994954 | 16.6 | 24905476 | 16.3 |
| CTRL5 | 31662194 | 22505190 | 71.1 | 10 | 3037353 | 21.2 | 3187360 | 19.3 | 15554509 | 18.4 |
| CTRL6 | 21421565 | 14757051 | 68.9 | 10 | 1868637 | 19.0 | 1919842 | 17.2 | 9197961 | 16.4 |
| CTRL7 | 41303233 | 26481247 | 64.1 | 10 | 2097839 | 29.7 | 2167716 | 26.3 | 10828729 | 24.5 |
| CTRL8 | 31946198 | 15597063 | 48.8 | 10 | 2512865 | 21.2 | 2602296 | 19.2 | 12787811 | 18.2 |
| CTRL9 | 31711354 | 18821446 | 59.4 | 10 | 1562349 | 30.8 | 1598420 | 26.7 | 7900159 | 24.4 |
| SELF1 | 28763651 | 18390361 | 63.9 | 10 | 1493876 | 27.1 | 1515800 | 23.5 | 7576974 | 21.7 |
| SELF2 | 36674050 | 22790592 | 62.1 | 10 | 1425135 | 31.1 | 1444072 | 26.6 | 7270183 | 24.4 |
| SELF3 | 34802179 | 24000531 | 69.0 | 10 | 2587463 | 22.4 | 2687407 | 20.2 | 13344706 | 19.2 |
| SELF4 | 31708014 | 15682626 | 49.5 | 10 | 1200599 | 19.3 | 1168634 | 17.2 | 5741833 | 16.2 |
| SELF5 | 23349007 | 14327361 | 61.4 | 10 | 1268181 | 16.4 | 1246853 | 15.0 | 5988118 | 14.4 |
| SELF6 | 38155463 | 25100747 | 65.8 | 10 | 2667406 | 19.2 | 2764073 | 17.5 | 13778258 | 17.0 |
| min | 21421565 | 14327361 | 48.8 | 10 | 1200599 | 16.4 | 1168634 | 15.0 | 5741833 | 14.4 |
| Max | 41303233 | 27986405 | 73.6 | 10 | 4527147 | 31.1 | 4994954 | 26.7 | 24905476 | 24.5 |
| Mean | 31807021 | 20349329 | 63.9 | 10 | 2185697 | 23.1 | 2269321 | 20.6 | 11207565 | 19.4 |

**Supplementary Figure S1.** **Histograms of DMR from treatment vs. control comparisons for all time points and methylation contexts.** Data refer to frequency distributions of methylation differences calculated throughout the genome.

**

**

**Supplementary Figure S2. Agarose gel electrophoresis of self-DNA samples used for treatment solution preparation.** Picture shows samples of nucleic acids extracted from A. thaliana fresh leaf material before (wells 1, 4 and 7) and after (2, 5 and 8) treatment with RNase A and after sonication (3, 6 and 9).


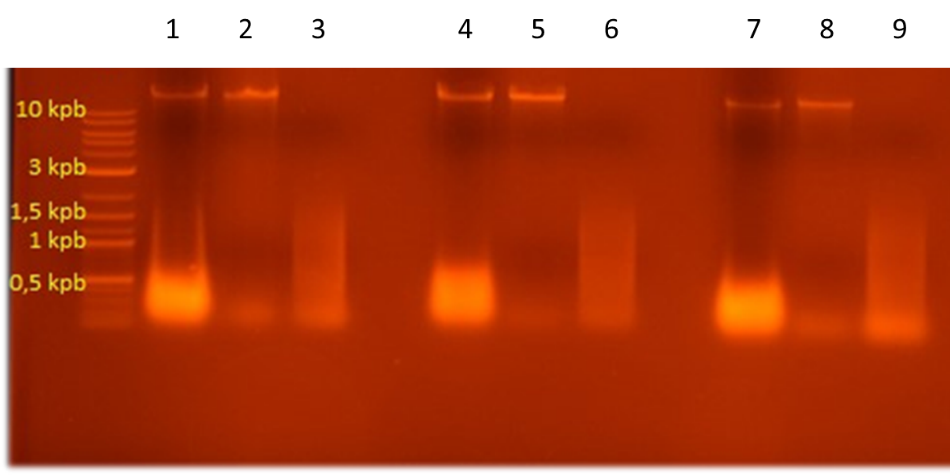

Supplement: Supplementary file 1 [file epigenomes-09-00013-s001.zip › Supplementary Tables & Figures.docx]
